# Supplementary figures and images for: Enhanced Protection of Biological Membranes during Lipid Peroxidation: Study of the Interactions between Flavonoid Loaded Mesoporous Silica Nanoparticles and Model Cell Membranes
Source: Int J Mol Sci. 2019 Jun 1;20(11):2709. doi: 10.3390/ijms20112709 (PMC6600359; doi:10.3390/ijms20112709)

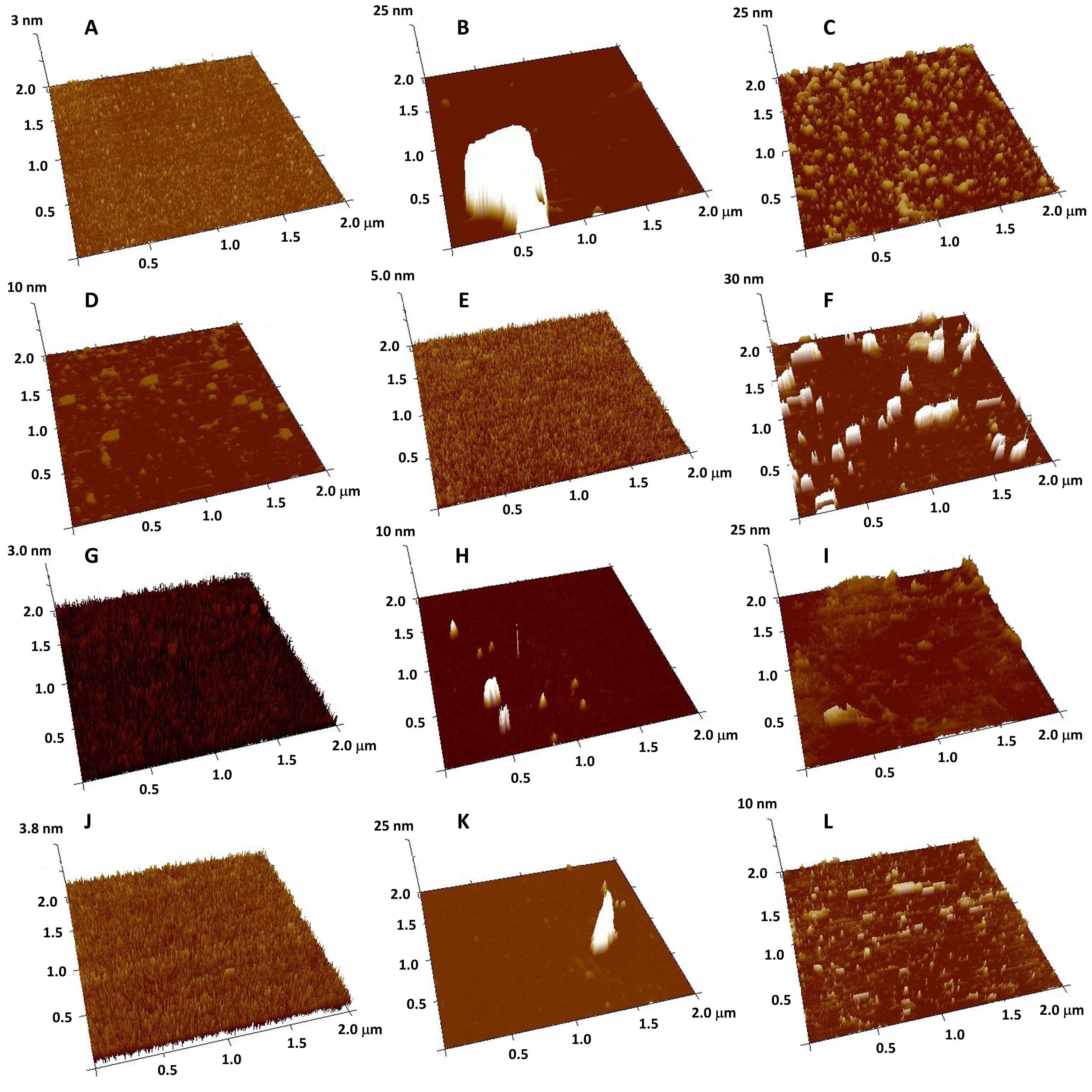

Supplement: Supplementary file 1 [file ijms-20-02709-s001.zip › Figure S1.jpg]

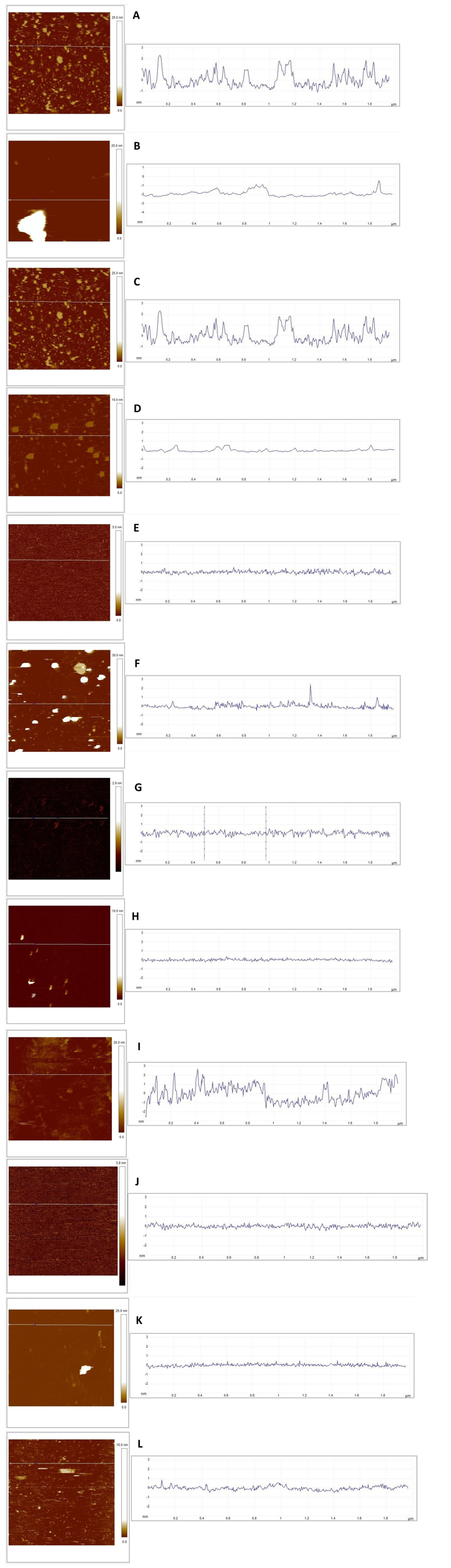

Supplement: Supplementary file 1 [file ijms-20-02709-s001.zip › Figure S2.jpg]

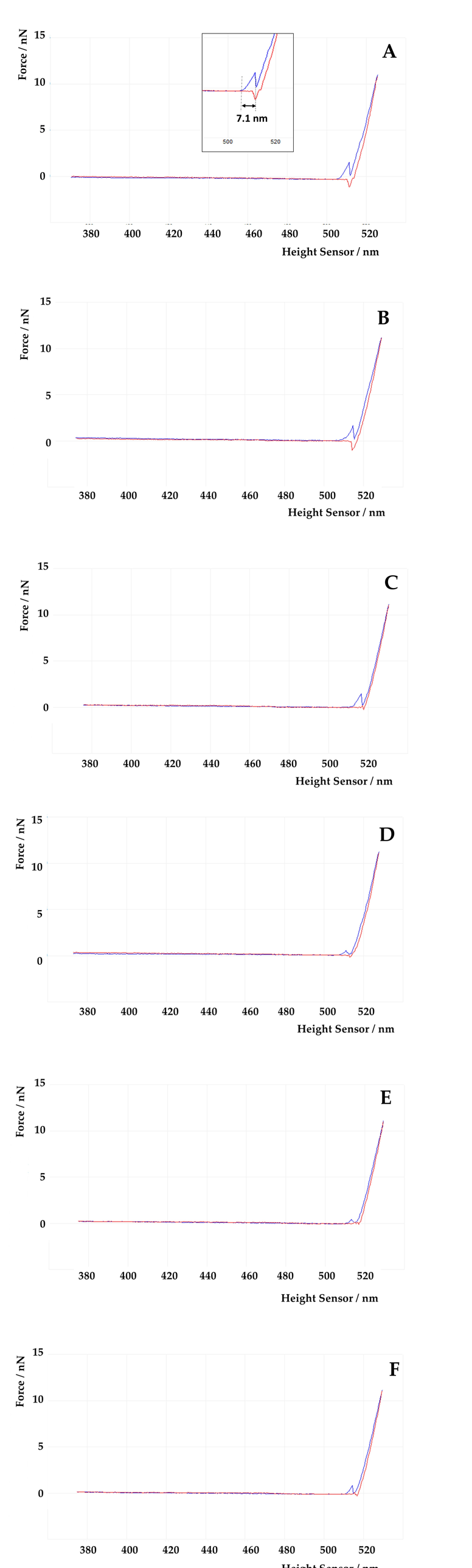

Supplement: Supplementary file 1 [file ijms-20-02709-s001.zip › Figure S3.jpg]

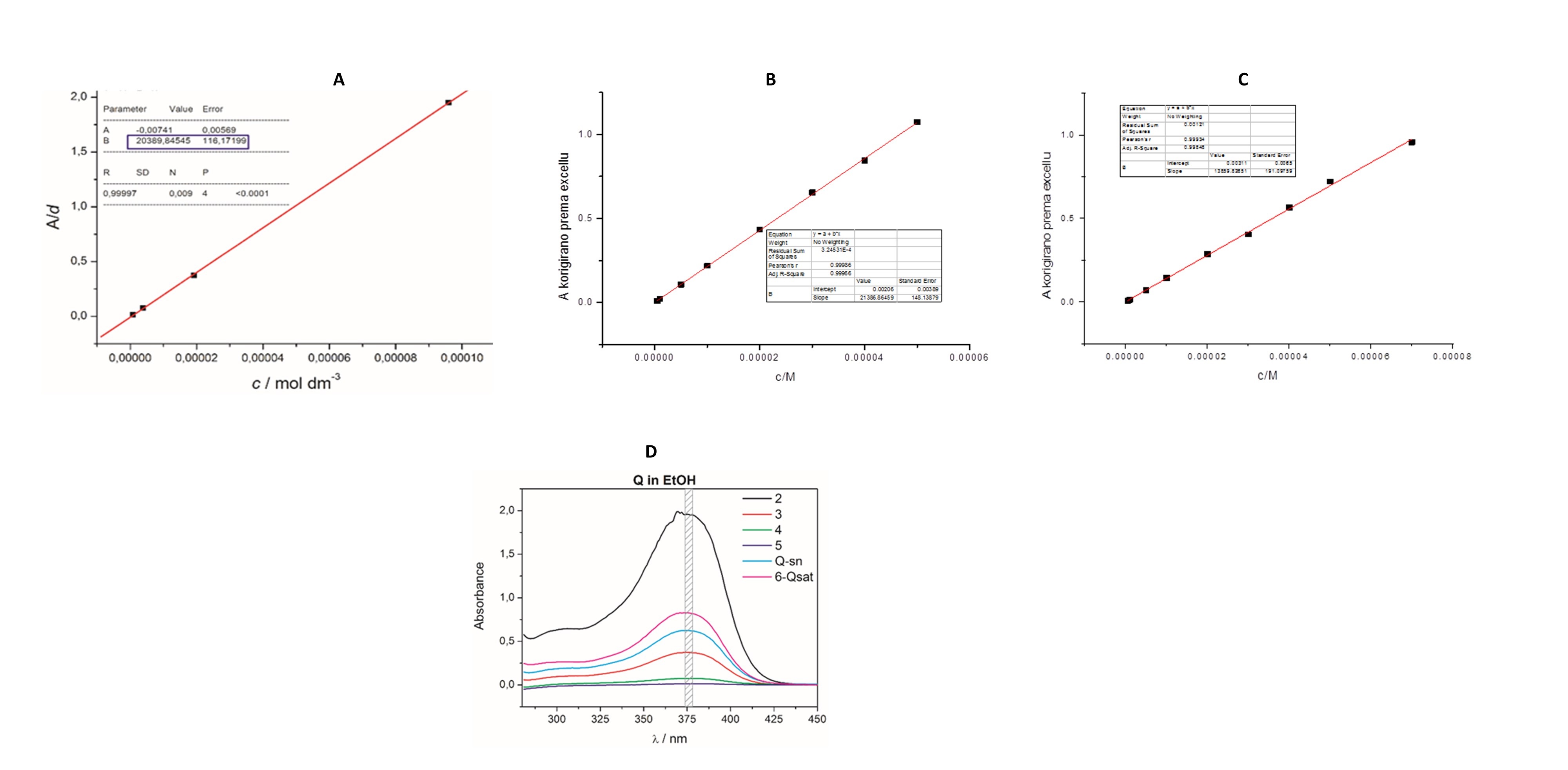

Supplement: Supplementary file 1 [file ijms-20-02709-s001.zip › Figure S4.jpg]
